# Emergence of azithromycin-resistant extensively drug-resistant *Salmonella* Kentucky ST314 in Taiwan

**DOI:** 10.3389/fmicb.2026.1832575

**Published:** 2026-06-01

**Authors:** Ying-Shu Liao, Yu-Ping Hong, You-Wun Wang, Shao-Chun Kuo, Ru-Hsiou Teng, Shiu-Yun Liang, Hsiao-Lun Wei, Jui-Hsien Chang, Chien-Shun Chiou

**Affiliations:** Center for Research, Diagnostics and Vaccine Development, Centers for Disease Control, Ministry of Health and Welfare, Taichung, Taiwan

**Keywords:** antimicrobial resistance, azithromycin, extensively drug-resistant, molecular epidemiology, multidrug-resistant, non-typhoidal *Salmonella*, resistance mechanism

## Abstract

*Salmonella enterica* serovar Kentucky (*S.* Kentucky) has emerged globally as a multidrug-resistant lineage but has historically been rare in Taiwan. Here, we report a recent surge of azithromycin-resistant extensively drug-resistant (AziR-XDR) *S.* Kentucky identified through nationwide surveillance from 2004 to 2025. Among 45,223 *Salmonella* isolates from humans, 192 (0.42%) were identified as *S.* Kentucky, with a marked increase observed in 2024─2025. Whole-genome sequencing of 165 isolates recovered from humans, animals, and chicken meat identified three major sequence types (ST314, ST198, and ST152), with ST314 accounting for 86.1% of the isolates. Thirty-two isolates were classified as AziR-XDR, including 28 ST314 and 4 ST198 isolates. Phylogenetic analysis revealed a dominant ST314 lineage carrying a conserved chromosomal multiple resistance region harboring *aadA1*, *aph(3′)-Ia*, *bla*_TEM-1_, *dfrA5*, *floR*, *sul3*, and *tet(A)* together with a *gyrA*_D87G mutation. AziR-XDR isolates arose when strains from this lineage further acquired a non-mobilizable IncFIB(pB171) plasmid carrying *bla*_DHA-1_, *mph(A)*, *qnrB*, and *sul1*. The observed phylogenetic diversity of ST314 isolates, together with structural variation in the IncFIB(pB171) plasmids, suggests that this resistance architecture had diversified before its introduction into Taiwan. In the NCBI Pathogen Detection database, the complete AziR-XDR resistance configuration has been observed only among Taiwanese ST314 isolates to date. Genomic comparisons further indicated close genetic relatedness between Taiwanese isolates and strains circulating in China and internationally. In contrast, ST198 AziR-XDR isolates were embedded within broader international lineages. These findings indicate that the recent increase of AziR-XDR ST314 in Taiwan is driven by a lineage carrying a pre-assembled resistance architecture and highlight the importance of genomic surveillance in tracking the emergence and dissemination of high-risk resistant clones.

## Introduction

*Salmonella enterica* is one of the leading causes of bacterial foodborne illness worldwide and remains a major public health concern due to its global distribution, zoonotic reservoirs, and substantial disease burden in humans (Majowicz et al., 2010; Kirk et al., 2015; GBD 2017 Non-Typhoidal Salmonella Invasive Disease Collaborators, 2019). In addition to its global impact, *Salmonella* poses an increasing clinical and public health challenge driven by the global emergence and dissemination of antimicrobial-resistant strains. Reflecting these concerns, the World Health Organization has designated fluoroquinolone-resistant *Salmonella* as high-priority pathogens for surveillance and control (WHO, 2024).

In Taiwan, salmonellosis is endemic, and antimicrobial resistance has emerged as a key epidemiological feature through sustained surveillance efforts (Liao et al., 2024). Over the past two decades, molecular epidemiological studies have demonstrated that antimicrobial resistance in *Salmonella* is largely driven by the emergence and sustained circulation of multidrug-resistant (MDR) clones (Chang et al., 2005; Hong et al., 2018; Chiou et al., 2019; Liao et al., 2019; Liao et al., 2025; Liao et al., 2023; Hong et al., 2026). These MDR clones have accumulated resistance to multiple clinically important antimicrobials, including fluoroquinolones, third-generation cephalosporins, and, more recently, azithromycin (Hong et al., 2026; Hong et al., 2021; Chiou et al., 2023).

*Salmonella* serovar Kentucky (*S.* Kentucky) has been recovered from humans, poultry, livestock, food products, and environmental sources, with poultry consistently identified as a major reservoir (Liu et al., 2024; Soltys et al., 2021; Samper-Cativiela et al., 2022). Genomic and multilocus sequence typing analyses indicate that *S.* Kentucky is polyphyletic. Its global epidemiology is dominated by sequence type ST198, a multidrug-resistant lineage widely disseminated and frequently associated with high-level fluoroquinolone resistance (Hawkey et al., 2019; Coipan et al., 2020; European Food Safety Authority, European Centre for Disease Prevention Control, 2025). Other lineages, including ST152 and ST314, have also been reported, with ST314 associated with multidrug resistance in Asia (Soltys et al., 2021; Haley et al., 2016; Chen et al., 2025).

Long-term nationwide surveillance in Taiwan since 2004 has consistently identified *S.* Kentucky as a rare serovar among human *Salmonella* isolates, accounting for only sporadic cases (Liao et al., 2024). However, recent surveillance has suggested a marked increase in both the absolute number and relative proportion of *S.* Kentucky isolates recovered from human clinical specimens during 2024–2025. This increase was accompanied by the emergence of extensively drug-resistant (XDR) isolates. Notably, some of these XDR isolates were also resistant to azithromycin, suggesting the emergence of azithromycin-resistant XDR (AziR-XDR) *S.* Kentucky in Taiwan. However, the genomic characteristics, population structure, and evolutionary origin of these recently emerging *S.* Kentucky lineages in Taiwan remain unclear.

In this study, we aimed to characterize the recent emergence of *S.* Kentucky in Taiwan by integrating nationwide surveillance data collected during 2004–2025 with whole-genome sequencing. We further sought to investigate the population structure, antimicrobial resistance determinants, and the potential origin of azithromycin-resistant XDR clones.

## Materials and methods

### Bacterial isolates

*Salmonella* isolates from human salmonellosis cases were obtained through PulseNet Taiwan, a national molecular surveillance network established in 2004 that collects isolates from collaborating hospitals across Taiwan. Human isolates were collected between 2004 and 2025. Confirmed salmonellosis was defined as the isolation of *Salmonella* from clinical specimens in patients presenting with clinical symptoms. This surveillance program was approved by the Institutional Review Board of the Taiwan Centers for Disease Control (Taiwan CDC) (IRB No.: 113107#1). Isolates from retail chicken meat were obtained from the Taiwan Food and Drug Administration in 2017 and from two investigation studies conducted by the Taiwan CDC in 2019 and 2022. Animal isolates were recovered from diseased chickens, pigs, and one duck from 2011 to 2024 through the Animal Disease Diagnostic Center, National Chiayi University, Taiwan. Species identification for all isolates was confirmed using the MALDI Biotyper (Bruker Daltonics GmbH, Bremen, Germany). Isolates collected between 2004 and 2024 were characterized using the standardized PulseNet pulsed-field gel electrophoresis (PFGE) protocol for *Salmonella* (Ribot et al., 2006). *Salmonella* serotypes of isolates collected between 2004 and 2024 were assigned through clustering of PFGE patterns with those in the Taiwan CDC *Salmonella* PFGE database containing isolates with known serotypes, as previously described (Chiou et al., 2015). For isolates collected in 2025, serotypes were determined using whole-genome sequencing–based methods with SISTR v1.1.3.

### Antimicrobial susceptibility testing

Antimicrobial susceptibility testing (AST) was performed using the EUVSEC3 Sensititre broth microdilution MIC panel (TREK Diagnostic Systems Ltd., Thermo Fisher Scientific, East Grinstead, United Kingdom), which includes 15 antimicrobial agents recommended for antimicrobial resistance monitoring in *Salmonella* under the European Union surveillance protocol. Minimum inhibitory concentration (MIC) results were interpreted primarily using Clinical and Laboratory Standards Institute (CLSI) breakpoints for *Salmonella* and *Shigella* spp., and, when unavailable, CLSI interpretative criteria for Enterobacterales (CLSI, 2025). These interpretative criteria covered amikacin, ampicillin, azithromycin, cefotaxime, ceftazidime, chloramphenicol, ciprofloxacin, colistin, gentamicin, meropenem, nalidixic acid, sulfamethoxazole, tetracycline, and trimethoprim. For tigecycline, no CLSI interpretative criteria are available for *Salmonella*; therefore, resistance was defined using the European Committee on Antimicrobial Susceptibility Testing (EUCAST) breakpoint, with MIC values >0.5 mg/L considered resistant (European Committee on Antimicrobial Susceptibility Testing, 2026). For ciprofloxacin, MIC values were interpreted according to CLSI criteria, with isolates classified as susceptible (MIC ≤0.06 mg/L), intermediate (MIC 0.125–0.5 mg/L), representing reduced susceptibility, and resistant (MIC ≥1 mg/L).

MDR strains were defined as isolates resistant to the traditional first-line antimicrobials ampicillin, chloramphenicol, and trimethoprim–sulfamethoxazole. XDR strains were defined, following the definition proposed for *S.* typhi, as isolates resistant to the three first-line antimicrobials in addition to a fluoroquinolone (e.g., ciprofloxacin) and a third-generation cephalosporin (e.g., cefotaxime or ceftriaxone) (Klemm et al., 2018). Because reduced ciprofloxacin susceptibility (MIC 0.125–0.5 mg/L) has been associated with adverse clinical outcomes, including prolonged fever clearance time and treatment failure (Kadhiravan et al., 2005; Slinger et al., 2004), isolates exhibiting reduced ciprofloxacin susceptibility together with resistance to the three first-line antimicrobials and either cefotaxime or ceftazidime were also classified as XDR. AziR-XDR strains were defined as XDR isolates that additionally exhibited resistance to azithromycin.

### Whole-genome sequencing and analysis

A total of 165 *S.* Kentucky isolates recovered between 2005 and 2025 were selected for whole-genome sequencing (WGS). Isolate selection was based on PFGE genotypes, with representative isolates chosen to capture diversity across different sources, time periods, and antimicrobial resistance profiles. Among isolates sharing the same PFGE genotype, at least one representative isolate was selected from each source category and, where possible, from different years. All 165 isolates were sequenced using the Illumina MiSeq platform (Illumina Inc., San Diego, CA, USA). In addition to isolates collected in 2025, seven isolates recovered before 2025 were selected for ONT long-read sequencing to enable complete genome assembly. Four of these isolates were classified as ST198 AziR-XDR, while the remaining three were recovered from chickens and chicken meat and carried the characteristic resistance determinants identified in human isolates, including the seven antimicrobial resistance genes [*aadA1*, *aph(3′)-Ia*, *bla*_TEM-1_, *dfrA5*, *floR*, *sul3*, and *tet(A)*] together with the *gyrA*_D87G mutation. These isolates were selected to confirm the genomic locations of resistance determinants, particularly to distinguish chromosomal and plasmid-borne elements.

Bacterial genomes were sequenced using the Illumina MiSeq platform (Illumina Inc., San Diego, CA, USA). WGS was performed at the Taiwan Centers for Disease Control, except for one isolate, which was sequenced by Dr. Achtman’s group at the Wellcome Sanger Institute and the University of Warwick. In addition, all isolates collected in 2025 and seven isolates recovered before 2025 were further sequenced using the Oxford Nanopore Technologies (ONT) platform (Oxford Nanopore Technologies Ltd., Oxford, UK) to generate long-read data for complete genome assembly.

Genomic DNA was extracted using the DNeasy Blood & Tissue Kit (cat. no. 69506; Qiagen, Hilden, Germany) according to the manufacturer’s instructions. Short-read libraries were prepared using the Illumina DNA Prep library preparation kit (cat. no. IL20018705; Illumina Inc., San Diego, CA, USA), which includes tagmentation of genomic DNA, bead-based cleanup, PCR amplification with adapters, and final purification. Illumina sequencing was performed on the MiSeq platform (Illumina Inc.) using a 2 × 300-bp paired-end configuration, and all isolates achieved >30 × coverage. Raw Illumina reads were processed using fastp^1^ for quality filtering and adapter trimming, and *de novo* genome assemblies were generated using SPAdes v4.0.0 (Bankevich et al., 2012). Contigs of low quality were removed based on length (<200 bp), sequencing coverage (<2×), or excessive homopolymer composition. Antimicrobial resistance determinants were identified using AMRFinderPlus v4.0.3 (Feldgarden et al., 2021). In silico serotype prediction was performed using SISTR v1.1.3,^2^ and plasmid incompatibility (replicon) types were identified using PlasmidFinder v.2.2.0.^3^ Multilocus sequence types (STs) were assigned using the mlst software tool v.2.25.0.^4^

Long-read sequencing was conducted on the MinION sequencing platform using R10.4.1 flow cells. Raw signal data (POD5 format) were basecalled using Dorado v1.1.1 with the SUP5.2.0 model to generate FASTQ reads. Long-read assemblies were constructed using Flye v2.9.6.^5^ Circular contigs were re-oriented using dnaapler v1.2.0^6^ and subsequently polished using Medaka v2.0.1.^7^

WGS data generated in this study have been deposited in the NCBI database, and the corresponding accession numbers are provided in Supplementary Table S1. To assess genomic relatedness with publicly available genomes, isolates were compared using the NCBI Pathogen Detection system,^8^ which assigns isolates to SNP clusters based on core-genome SNP (cgSNP) analysis.

### Phylogenetic analysis

Core genome SNP (cgSNP) analysis was performed using a reference-based pipeline. Illumina reads were mapped to the complete genome sequence of *S.* Kentucky strain R25.0102 (GenBank accession: CM137136.1) using Snippy^9^ to identify SNPs and generate a core genome SNP alignment. Recombinant regions were identified and removed using Gubbins,^10^ resulting in a recombination-filtered SNP alignment. The filtered SNP alignment was used to infer a maximum-likelihood phylogenetic tree with RAxML-NG,^11^ implemented within the Gubbins pipeline, under the GTRGAMMA nucleotide substitution model. No bootstrap replicates were performed. The resulting tree was midpoint-rooted and visualized using Interactive Tree of Life (iTOL) v7.^12^

### Conjugation assay

Conjugation assays were performed using *S.* Kentucky R25.0102 and R25.1404 as donor strains, and *S.* Kentucky SD14.100, R16.1211, R19.0552, and *Escherichia coli* C600 as recipient strains. Donors were maintained on LB agar supplemented with cefotaxime (2 mg/L) to ensure maintenance of resistance determinants, while recipients were selected on LB agar containing rifampicin (100 mg/L) to select for rifampicin-resistant recipients. Single colonies of each donor and rifampicin-resistant recipient were cultured in LB broth at 37 °C with shaking at 200 rpm for 6–8 h. Donor and recipient cultures were mixed at a 1:10 ratio, spread onto antibiotic-free LB agar plates, and incubated overnight at 37 °C. Bacterial cells were then resuspended in PBS, serially diluted, and plated onto LB agar containing cefotaxime (2 mg/L) and rifampicin (100 mg/L) to select for transconjugants.

## Results

### Epidemiological trends of *Salmonella* Kentucky in human salmonellosis

From 2004 to 2025, a total of 45,223 *Salmonella* isolates were collected from hospitals through a long-term surveillance project, among which 192 (0.42%) were identified as *S.* Kentucky (Supplementary Table S2). Between 2004 and 2013, *S.* Kentucky was rarely detected, with only a single isolate identified in 2005. Since 2014, *S.* Kentucky has been consistently detected in surveillance and has shown an increasing trend over time. The annual proportion of *S.* Kentucky among all *Salmonella* isolates rose from 0.22% in 2014 to 4.16% in 2025 (Supplementary Table S2).

**Figure 1:**
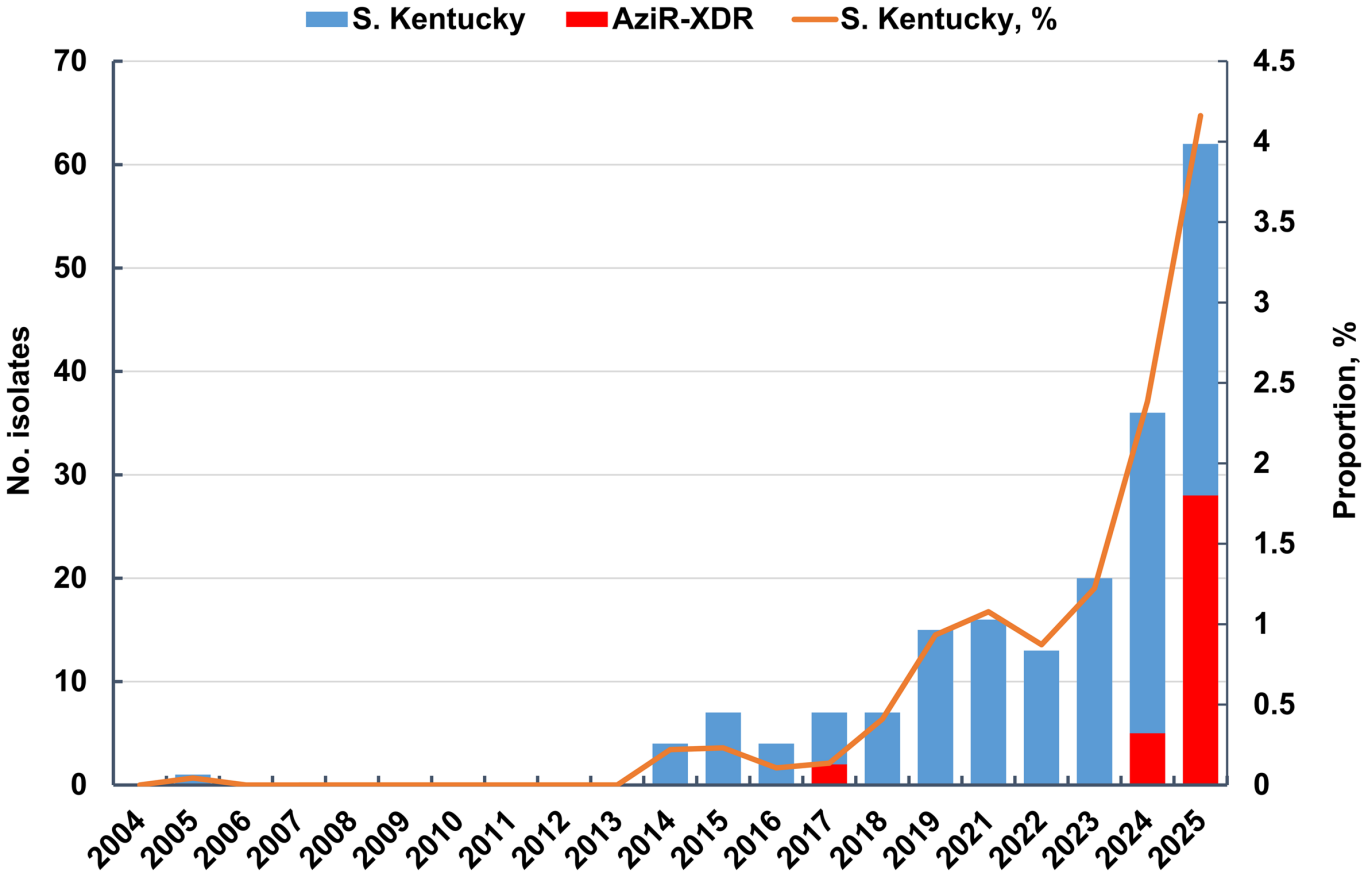
Annual distribution of *Salmonella enterica* serovar Kentucky isolates from human salmonellosis cases in Taiwan, 2004–2025. Red bars indicate azithromycin-resistant extensively drug-resistant (AziR-XDR) isolates, and the line represents the proportion of *S.* Kentucky among all human *Salmonella* isolates each year. Among the five AziR-XDR isolates detected in 2024, four were ST314 isolates with identical PFGE patterns and antimicrobial susceptibility profiles, but only one isolate underwent whole-genome sequencing.

Notably, the increase in *S.* Kentucky became particularly pronounced in 2024 and 2025, during which both the absolute number of isolates and their proportion among all *Salmonella* isolates nearly doubled compared with the preceding years (Figure 1). In parallel, AST revealed that nearly half (45.2%) of the *S.* Kentucky isolates collected in 2025 met the criteria for XDR and exhibited azithromycin resistance (AziR-XDR). Retrospective analysis indicated that AziR-XDR *S.* Kentucky isolates were sporadically detected as early as 2017 (*n* = 2) and again in 2024 (*n* = 5), before their marked increase in 2025 (*n* = 28) (Figure 1).

### Genetic lineages of *Salmonella* Kentucky

PFGE-based cluster analysis of 267 *S.* Kentucky isolates from humans and non-human sources collected between 2005 and 2024 identified three distinct clusters, designated clusters I, II, and III (Supplementary Figure S1). Subsequent whole-genome sequencing demonstrated that isolates in PFGE cluster I corresponded to sequence type ST198, whereas those in cluster II corresponded to ST314 and those in cluster III corresponded to ST152. For isolates collected in 2025, PFGE was not performed, and cluster assignment was inferred based on WGS-derived sequence types. Specifically, isolates identified as ST198 and ST314 were assigned to PFGE clusters I and II, respectively, and were included in the temporal analysis (Supplementary Table S2). Among human isolates, ST198 strains were detected sporadically throughout the study period and accounted for 9.9% (19/192) of all *S.* Kentucky isolates (Supplementary Table S2). In contrast, ST314 strains were first detected in 2014, rapidly became the predominant lineage thereafter, and accounted for 90.1% (173/192) of *S.* Kentucky isolates collected between 2004 and 2025. No ST152 strains were identified among human isolates.

Genomic characterization of 165 *S.* Kentucky isolates collected from humans (*n* = 116), chickens (*n* = 29), chicken meat (*n* = 16), pigs (*n* = 3), and a duck was conducted (Supplementary Table S1). cgSNP-based phylogenetic analysis inferred using a maximum-likelihood approach revealed that these isolates belonged to three genetically distinct lineages, corresponding to sequence types ST152, ST198, and ST314 (Supplementary Figure S2).

Among these three lineages, ST314 was the predominant genotype, accounting for 86.1% (142/165) of the isolates, and was identified across multiple sources, including humans, chickens, chicken meat, and pigs (Supplementary Table S3). ST198 represented 11.5% (19/165) of the isolates and was detected sporadically in human cases throughout the study period, as well as in isolates from chickens, chicken meat, and a duck. The earliest *S.* Kentucky isolate identified in this study, recovered from a human case in 2005, was ST198. ST152 constituted the remaining 2.4% of isolates (4/165) and was restricted to poultry-associated isolates, being identified exclusively in chickens.

### Antimicrobial resistance in *Salmonella* Kentucky

Antimicrobial resistance inferred from resistance determinants in the 165 isolates revealed a substantial burden of multidrug resistance. Overall, 85.5% (141/165) of isolates met the MDR definition, including 35.2% (58/165) classified as XDR and 19.4% (32/165) as AziR-XDR (Table 1). Both ST314 and ST198 isolates exhibited high MDR rates of 94.4% (134/142) and 41.2% (7/17), respectively, whereas no inferred MDR phenotype was observed among ST152 isolates. Notably, the majority of XDR and AziR-XDR isolates belonged to ST314.

**Table 1:** Antimicrobial resistance profiles and associated resistance determinants among 165 *Salmonella enterica* serovar Kentucky isolates.

Category/Antimicrobial*	No. isolates with inferred resistance				ARGs	Genotypic resistance rate (%)	Phenotypic resistance rate (%)
	Total (*n* = 165)	ST152 (*n* = 6)	ST198 (*n* = 17)	ST314 (*n* = 142)			
MDR	141	0	7	134	—	85.5	84.8
XDR	58	0	4	54	—	35.2	35.2
AziR-XDR	32	0	4	28	—	19.4	19.4
Amikacin	3	0	3	0	*aph(3′)-VI, rmtB1*	1.8	0.7
Ampicillin	152	6	9	137	*bla*_CMY-16_ *, bla*_CMY-2_ *, bla*_CTX-M-55_ *, bla*_DHA-1_ *, bla*_NDM-1_ *, bla*_OXA-48_ *, bla*_TEM-1_ *, bla*_TEM-176_ *, bla*_TEM-215_	92.1	92.1
Azithromycin	34	0	5	29	*erm(B), mph(A), mph(E), msr(E), ramAp*	20.6	20.6
Cefotaxime	61	1	5	55	*bla*_CMY-16_ *, bla*_CMY-2_ *, bla*_CTX-M-55_ *, bla*_DHA-1_ *, bla*_NDM-1_ *, bla*_OXA-48_	37.0	37.0
Ceftazidime	61	1	5	55	*bla*_CMY-16_ *, bla*_CMY-2_ *, bla*_CTX-M-55_ *, bla*_DHA-1_ *, bla*_NDM-1_ *, bla*_OXA-48_	37.0	37.0
Chloramphenicol	154	5	11	138	*catA2, cmlA1, floR, ramAp*	93.3	93.3
Ciprofloxacin^NS^	138	1	7	130	*gyrA*_D87G, *gyrA*_D87N, *gyrA*_S83F, *parC*_S80I, *qnrB, qnrS1, ramAp*	83.6	83.6
Colistin	0	0	0	0	—	0	0
Gentamicin	18	1	4	13	*aac(3)-Id, aac(3)-IId, aac(3)-Iva, rmtB1*	10.9	10.9
Meropenem	3	0	3	0	*bla*_NDM-1_ *, bla*_OXA-48_	1.8	1.2
Nalidixic acid	137	1	6	130	*gyrA*_D87G, *gyrA*_D87N, *gyrA*_S83F, *parC*_S80I, *ramAp*	83.0	83.0
Sulfamethoxazole	152	1	13	138	*ramAp, sul1, sul2, sul3*	92.1	92.1
Tetracycline	152	5	12	135	*ramAp, tet(A), tet(B), tet(M)*	92.1	92.1
Tigecycline	1	0	1	0	*ramAp*	0.6	25.6
Trimethoprim	146	0	11	135	*dfrA12, dfrA14, dfrA17, dfrA5, dfrA51, ramAp*	88.5	88.4
Cefoxitin	60	1	4	55	*blaCMY, bla*_CMY-16_ *, bla*_CMY-2_ *, bla*_DHA-1_ *, bla*_NDM-1_ *, ramAp*	36.4	NA
Clindamycin	15	0	5	10	*erm(B), lnu(F)*	9.1	NA
Erythromycin	35	0	5	30	*erm(B), estT, mph(A), mph(E), msr(E)*	21.2	NA
Fosfomycin	3	0	3	0	*fosA3*	1.8	NA
Hygromycin B	4	1	0	3	*aph(4)-Ia*	2.4	NA
Kanamycin	140	0	7	133	*aac(6′)-Ib3, aph(3′)-Ia, aph(3′)-IIa, aph(3′)-VI*	84.8	NA
Rifampicin	3	0	3	0	*arr-2*	1.8	NA
Streptomycin	149	5	13	131	*aadA1, aadA2, aadA22, aadA5, aadA7, aph(3″)-Ib, aph(6)-Ic, aph(6)-Id*	90.9	NA

Genotypic resistance rate indicates the proportion of isolates carrying resistance determinants. Phenotypic resistance rate indicates resistance determined by antimicrobial susceptibility testing. NS (non-susceptible) includes both resistant and intermediate isolates.

For the traditional first-line agents, resistance determinants were highly prevalent for ampicillin (92.1%), chloramphenicol (93.3%), sulfamethoxazole (92.1%), and trimethoprim (88.5%). Resistance determinants to third-generation cephalosporins were also frequently identified, with inferred resistance to cefotaxime detected in 37.0% (61/165) of isolates, primarily mediated by AmpC *β*-lactamases including *bla*_CMY-2_ and *bla*_DHA-1_.

Resistance to fluoroquinolones was common. Inferred non-susceptibility to ciprofloxacin was detected in 83.6% (138/165) of isolates, and nalidixic acid resistance in 83.0% (137/165). Nalidixic acid resistance was mainly associated with mutations in *gyrA* and *parC*, whereas ciprofloxacin resistance was mediated by these mutations either alone or in combination with *qnrB* and *qnrS1*. In addition, high proportions of isolates carried resistance determinants to kanamycin (84.8%), streptomycin (90.9%), and tetracycline (92.1%).

Of particular clinical concern, resistance determinants associated with azithromycin and carbapenem resistance, which are considered last-resort therapies for XDR *S. typhi* infections, were also detected. Azithromycin resistance determinants were identified in 34 isolates (20.6%), all carrying *mph(A)*, with four isolates additionally harboring *erm(B)*, *mph(E)*, *msr(E)*, and *ramAp*. Carbapenem resistance determinants were detected in three isolates (1.8%), including two carrying *bla*_NDM-1_ and one carrying *bla*_OXA-48_.

Comparison of genotypic resistance predictions with AST data showed high concordance across most antimicrobials. For the 15 antimicrobials tested, inferred resistance rates were largely consistent with phenotypic resistance rates (Table 1), with a few discrepancies. For amikacin, resistance was inferred in three isolates based on the presence of *aph(3′)-VI* or *rmtB1*, whereas only one isolate showed phenotypic resistance. Two isolates carrying *aph(3′)-VI* contained upstream deletions affecting the promoter region. For meropenem, three isolates carried carbapenem resistance determinants. Phenotypic resistance was observed in two isolates carrying *bla*_NDM-1_, whereas the isolate carrying *bla*_OXA-48_ showed an elevated MIC but remained below the clinical resistance breakpoint. For tigecycline, the phenotypic resistance rate (25.6%) exceeded the genotypic resistance rate (0.6%). Although 42 isolates were classified as tigecycline-resistant based on the applied breakpoint, their MIC values were relatively low (1–2 mg/L). Notably, 39 of these isolates carried *tet(A)*. Tet(A) variants or overexpression of *tet(A)* have been reported to contribute to increased tigecycline MICs (Chiu et al., 2017; Zou et al., 2024).

### Phylogenetic analysis of ST314 isolates

**Figure 2:**
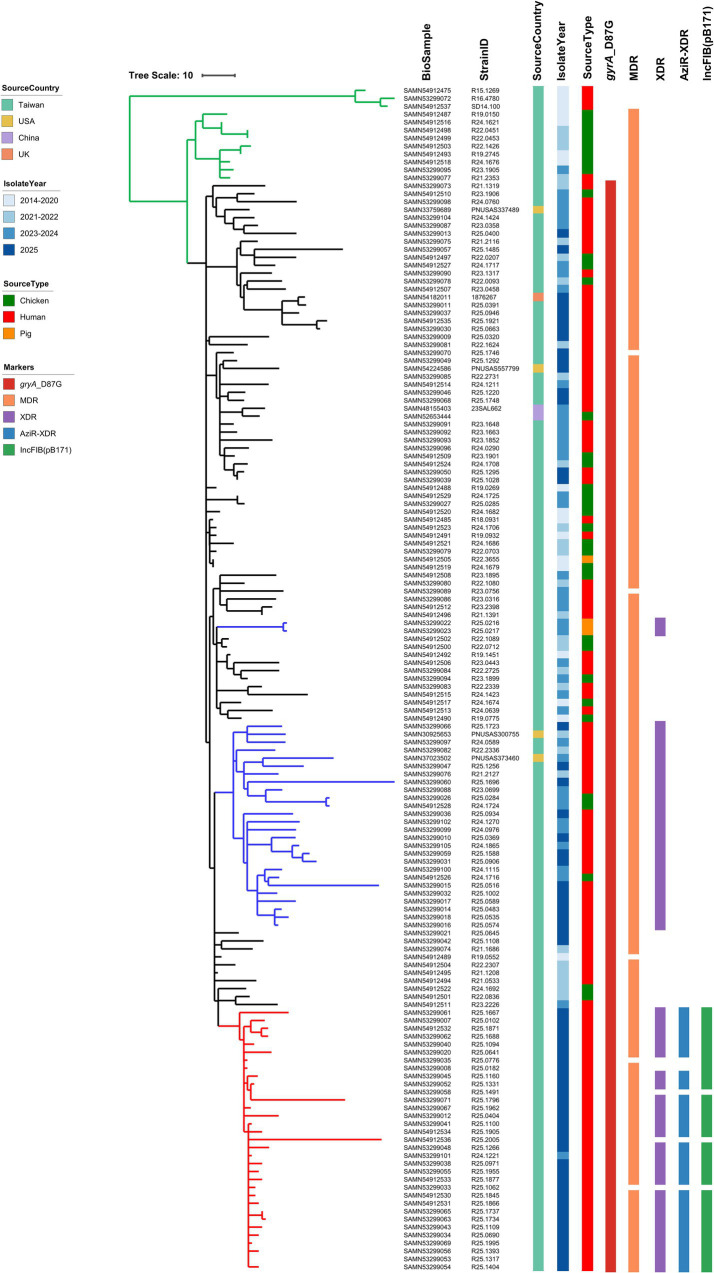
cgSNP-based phylogenetic tree of 149 ST314 *Salmonella enterica* serovar Kentucky isolates. The maximum-likelihood tree was inferred from cgSNP profiles to assess genetic relatedness among isolates. Scale bar indicates the number of SNP differences.

cgSNP-based phylogenetic analysis of 149 ST314 *S.* Kentucky isolates, including 142 from Taiwan and 7 from other countries—the USA (*n* = 4; SAMN30925653, SAMN33759689, SAMN37023502, SAMN54224586), China (*n* = 2; SAMN48155403, SAMN52653444), and the UK (*n* = 1; SAMN54182011)—retrieved from the NCBI database, revealed generally short branch lengths across the phylogeny (Figure 2). A major lineage comprising 137 isolates was identified and was characterized by the presence of a multiple resistance region (MRR) together with the *gyrA*_D87G mutation. Among these isolates, 120 carried an MRR containing seven antimicrobial resistance genes (ARGs), including *aadA1*, *aph(3′)-Ia*, *bla*_TEM-1_, *dfrA5*, *floR*, *sul3*, and *tet(A)*, whereas 17 isolates lacked one or more of these genes. A minor lineage consisting of 12 isolates lacked both the MRR and the *gyrA*_D87G mutation, as indicated by green branches in Figure 2. Within this lineage, three isolates were separated from the remaining nine isolates by relatively longer branches. Branches representing isolates carrying the IncFIB(pB171) plasmid are indicated in red, although this subcluster also includes three isolates lacking the plasmid. Branches colored in blue represent *bla*_CMY-2_-carrying XDR isolates. Isolates from chickens, pigs, and chicken meat were distributed across both major and minor lineages. All international isolates clustered within the major lineage and carried both the MRR and the *gyrA*_D87G mutation. Two isolates from the USA carried *bla*_CMY-2_ and were located within the *bla*_CMY-2_–carrying XDR subcluster.

### Genomic characteristics of ST314 AziR-XDR isolates

**Figure 3:**
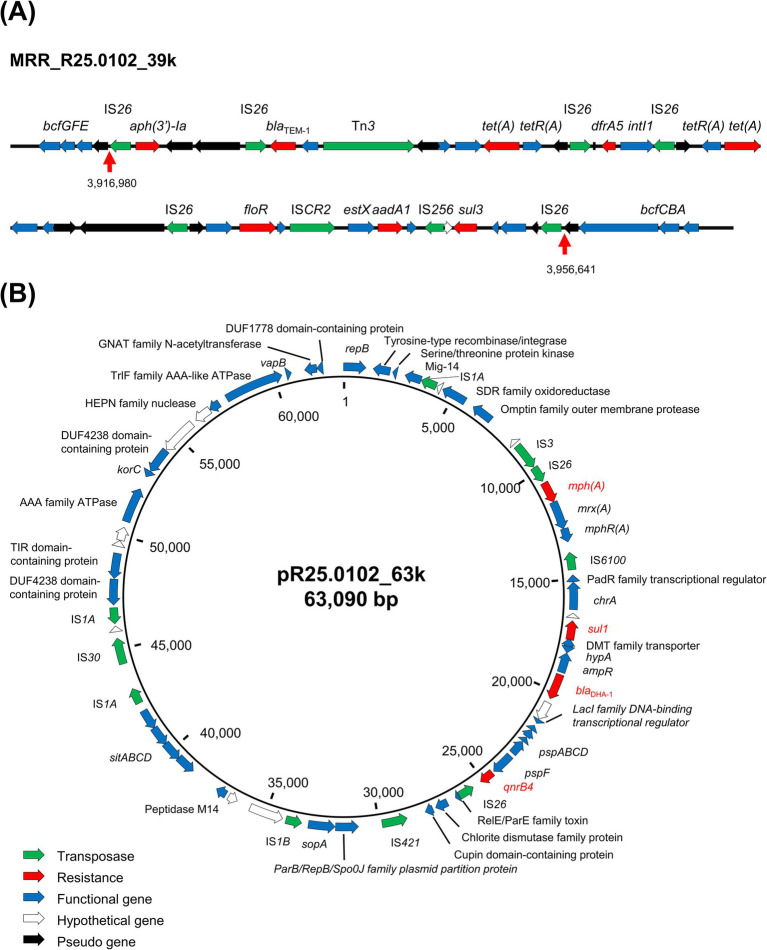
Genetic structure of the multiple resistance region MRR_R25.0102_39k **(A)** and circular plasmid pR25.0102_63k **(B)**. Arrows indicate predicted open reading frames and their transcriptional orientation. Antimicrobial resistance genes are highlighted in red, mobile genetic elements in green. MRR_R25.0102_39k is flanked by IS26 and inserted into *bcfD*, as indicated by the vertical red arrows. In plasmid pR25.0102_63k, resistance genes *mph(A)*, *sul1*, *bla*_DHA-1_, and *qnrB4* are located within a region flanked by IS26.

Among the 165 *S.* Kentucky isolates, 32 were classified as AziR-XDR, including 28 ST314 isolates and four ST198 isolates, all recovered from human clinical samples. Complete genome assembly showed that resistance determinants in ST314 AziR-XDR isolates were located on both the chromosome and an IncFIB(pB171) plasmid. As illustrated in Figure 3A, a ~ 39-kb (39,662 bp) MRR, designated MRR_R25.0102_39k based on isolate R25.0102, carried seven ARGs, *aadA1*, *aph(3′)-Ia*, *bla*_TEM-1_, *dfrA5*, *floR*, *sul3*, and *tet(A)*. This region was flanked by IS26 elements and inserted into *bcfD*, encoding a fimbrial protein, generating an 8-bp target site duplication (GTCTTAGG). Among the 28 ST314 AziR-XDR isolates, 24 carried the complete set of seven resistance genes, whereas four isolates lacked one or more genes within the MRR.

Complete genome assembly of R25.0102 identified a 63-kb (63,090 bp) IncFIB(pB171) plasmid, designated pR25.0102_63k, carrying four resistance genes, *bla*_DHA-1_, *mph(A)*, *qnrB*, and *sul1*, within a region flanked by IS26 elements but without an associated 8-bp target site duplication (Figure 3B). The combination of chromosomal and plasmid resistance determinants was consistent with an inferred AziR-XDR phenotype, conferring resistance to ampicillin, chloramphenicol, trimethoprim–sulfamethoxazole, third-generation cephalosporins (e.g., cefotaxime), fluoroquinolones (e.g., ciprofloxacin), and azithromycin.

The IncFIB(pB171) plasmid was detected in 30 of the 142 ST314 isolates. One plasmid variant identified in R25.0182 lacked the entire four ARGs. Comparative analysis using BRIG, with pR25.0102_63k as the reference, showed sequence variation among these plasmids (Supplementary Figure S3). Ten distinct plasmid patterns were identified among the 30 plasmids. Most variation occurred within the plasmid backbone between approximately 33 and 53 kb, whereas the resistance region was largely conserved except in the variant from R25.0182.

Analysis using the MOB-suite predicted that the IncFIB(pB171) plasmid would be non-mobilizable. Conjugation experiments using two donor strains, three pan-susceptible *S.* Kentucky recipient strains, and *Escherichia coli* C600 did not result in plasmid transfer.

### Genomic characteristics of ST198 AziR-XDR isolates

Four AziR-XDR isolates belonged to the ST198 lineage. All carried more than 15 ARGs and harbored the *gyrA*_D87N, *gyrA*_S83F, and *parC*_S80I mutations (Supplementary Table S1).

Isolates R25.0658 and R24.1333 showed similar genomic organization of resistance determinants. In R25.0658, ARGs and *ramAp* were located in two chromosomal MRRs, designated MRR_R25.0658_66k (66,782 bp) and MRR_R25.0658_18k (18,554 bp) (Supplementary Figure S4). MRR_R25.0658_66k was flanked by IS26 elements and inserted within the *dsbA* gene, encoding a DsbA family protein, with an 8-bp target site duplication (GCCACCAC). This region carried 12 ARGs, including *aac(3)-IId*, aadA2, *aph(3′)-Ia*, *arr-2*, *bla*_CTX-M-55_, *bla*_TEM-1_, dfrA14, *floR*, *fosA3*, *lnu(F)*, *mph(A)*, and *rmtB1*, together with *ramAp*. The second region, MRR_R25.0658_18k, was also flanked by IS26 but lacked an identifiable 8-bp target site duplication and contained *sul1*, *tet(A)*, and *aadA7*.

In R24.1333, resistance determinants were also located in two chromosomal MRRs, designated MRR_R24.1333_61k and MRR_R24.1333_24k. These regions together carried *aac(3)-IId*, *arr-2*, *bla*_TEM-1_, *dfrA14*, *floR*, *lnu(F)*, *bla*_DHA-1_, *mph(A)*, *qnrS1*, *ramAp*, *sul1*, and *tet(A)* (Supplementary Figure S5). In addition, *bla*_OXA-48_ was located on a 7,872-bp Col156 plasmid, designated pR24.1333_7.8 k (Supplementary Figure S6). BLAST searches against the NCBI nucleotide database (last accessed March 9, 2026) identified 10 highly similar Col156 *bla*_OXA-48_–carrying plasmids from *Escherichia coli*, *Klebsiella pneumoniae*, and *Salmonella* spp. (Supplementary Table S4). Nine of these plasmids were identical in size (7,872 bp) to pR24.1333_7.8 k.

The remaining two ST198 AziR-XDR isolates, R17.5342 and R17.5432, showed highly similar resistance profiles. Both carried *aac(6′)-Ib3*, *aadA5*, *aph(3″)-Ib*, *aph(3′)-V*I, *aph(6)-Id*, *bla*_CMY-16_, *bla*_NDM-1_, *dfrA17*, *erm(B)*, *floR*, *fosA3*, *mph(A)*, *mph(E)*, *msr(E)*, *sul1*, *sul2*, and *tet(A)* on a 183-kb (183,391 bp) IncC plasmid (Supplementary Figure S7).

### Global genomic context of AziR-XDR isolates

Comparison with publicly available genomes in the NCBI Pathogen Detection database indicated that ST314 AziR-XDR isolates from this study (e.g., R24.1221) were assigned to SNP cluster PDS000072600.66, comprising 463 genomes. Of these, 38.9% were from China and 29.4% from Taiwan. Within this cluster, 122 isolates carried the same seven resistance genes present in the MRR_R25.0102_39k and also harbored the *gyrA*_D87G mutation. Among these 122 isolates, 115 were collected in Taiwan, four were reported by the US Centers for Disease Control and Prevention (SAMN30925653, SAMN37023502, SAMN33759689, and SAMN54224586), two were from China (SAMN48155403 and SAMN52653444), and one was reported by the UK Health Security Agency (SAMN54182011). However, none of the non-Taiwan isolates within this SNP cluster simultaneously carried the complete set of resistance determinants for defining the AziR-XDR phenotype observed in this study.

Among the four ST198 AziR-XDR isolates, two isolates (R17.5342 and R17.5432) were assigned to a large SNP cluster PDS000251222.8, comprising 2,904 genomes from at least 53 countries. These two isolates shared an identical resistance gene profile with five very closely related isolates, including four isolates (SAMN31715374, SAMN16364189, SAMN20606958, and SAMN31209104) reported by the UK Health Security Agency and one (SAMN31058983) from the Microbiological Diagnostic Unit Public Health Laboratory, Australia. All seven AziR-XDR isolates carried an identical set of antimicrobial resistance genes, including *bla*_NDM-1_, consistent with carbapenem resistance.

Isolate R25.0658 was assigned to the NCBI Pathogen Detection SNP cluster PDS000251218.4, which comprised 152 genomes. Among these, 95.4% (145/152) isolates were recovered in China. The Chinese isolates were reported from multiple provinces and municipalities, including Henan, Shandong, Jiangsu, Anhui, Gansu, Liaoning, Beijing, and Yunnan. Many genomes in this cluster carried resistance determinants similar to those identified in R25.0658. Previous genomic analysis by Jiang et al. (2023) showed that several *S.* Kentucky isolates shared a chromosomal insertion of an IS26-bounded MRR at an identical site downstream of the *bcfABCDEFG* fimbrial gene cluster, interrupting a *dsbA* gene and generating identical 8-bp direct repeats.

## Discussion

In this study, we identified a clear epidemiological shift of *S.* Kentucky in Taiwan, characterized by the recent emergence and rapid increase of AziR-XDR strains. After remaining rare for more than a decade, *S.* Kentucky increased markedly in both incidence and relative prevalence during 2024–2025 (Figure 1). This increase was accompanied by a concurrent rise in XDR and AziR-XDR phenotypes (Table 1). Together, these findings indicate that the observed trend is not a background fluctuation but reflects a lineage-driven change in the population structure.

ST314 was the predominant lineage among recent *S.* Kentucky isolates (Supplementary Table S2). The cgSNP phylogeny showed limited genetic diversity, with short branch lengths and a compact clustering pattern (Figure 2). This pattern is consistent with the recent clonal expansion of a closely related lineage. In contrast, additional features indicate that this lineage had already undergone prior diversification. Several closely related subclusters were observed within ST314 (Figure 2). Multiple IncFIB(pB171) plasmid variants were also identified primarily among ST314 AziR-XDR isolates (Supplementary Figure S3). These findings suggest that the lineage had accumulated genetic variation over a period longer than the short time frame of its recent increase in Taiwan. Together, these observations support a scenario in which a previously diversified lineage was introduced and subsequently expanded.

Phylogenetic and comparative genomic analyses indicate that the major *S.* Kentucky lineages detected in Taiwan are not confined to Taiwan. ST314 isolates from Taiwan were assigned to an SNP cluster (PDS000072600.66) in the NCBI Pathogen Detection database that includes isolates from multiple countries. Within this cluster, a substantial proportion of isolates originated from China (38.9%), followed by Taiwan (29.4%). ST314 has been reported as prevalent in Asia, particularly in China (Chen et al., 2025; Wang et al., 2021; Gu et al., 2020). These observations support an external origin of ST314, rather than *de novo* emergence in Taiwan.

In contrast, the four AziR-XDR ST198 isolates identified in this study showed distinct links to international transmission networks. Three isolates were assigned to a large SNP cluster (PDS000251222.8) comprising genomes from at least 53 countries. Two of these isolates carried *bla*_NDM-1_ and exhibited carbapenem resistance, and shared identical resistance gene profiles with closely related isolates reported in the UK and Australia. The remaining isolate was assigned to another SNP cluster (PDS000251218.4) composed predominantly of isolates from China (95.4%), and shared resistance determinant profiles and conserved chromosomal insertion sites with multiple isolates reported from different regions in China (Jiang et al., 2023).

The AziR-XDR phenotype in ST314 appears to be driven by a pre-assembled resistance architecture rather than stepwise or random acquisition of individual determinants. In most ST314 isolates, a conserved chromosomal MRR together with the *gyrA*_D87G mutation formed a stable multidrug-resistant backbone (Figure 3A). This backbone was complemented by an IncFIB(pB171) plasmid carrying *bla*_DHA-1_, *mph(A)*, *qnrB*, and *sul1* (Figure 3B). Notably, plasmid-carrying and plasmid-lacking isolates coexisted within the same phylogenetic lineage (Figure 2), and comparative analysis showed structural variation among IncFIB(pB171) plasmids (Supplementary Figure S3). In addition, conjugation assays and in silico prediction indicated that this plasmid is non-mobilizable, with no evidence of transfer under the tested conditions. These observations do not support ongoing horizontal dissemination of resistance determinants. Instead, they are consistent with a model in which a composite resistance configuration was established prior to expansion and subsequently propagated through clonal spread.

Long-term surveillance in Taiwan has documented a sustained and increasing burden of antimicrobial resistance among *Salmonella* isolates (Liao et al., 2024). Building on these surveillance data, our previous investigations have documented the emergence and rapid expansion of MDR or XDR clones across multiple serovars, including *S.* Anatum (Chiou et al., 2019), *S.* Goldcoast (Liao et al., 2019), *S.* Agona (Liao et al., 2025), *S.* Infantis (Liao et al., 2023), and AziR-XDR *S.* Typhimurium (Hong et al., 2026). Collectively, these studies indicate that the repeated emergence of high-risk antimicrobial-resistant clones represents a persistent and recurring pattern in Taiwan. The present study adds AziR-XDR *S.* Kentucky as a recent example. Its temporal increase (Figure 1), clonal expansion (Figure 2), and defined resistance architecture (Figure 3) are consistent with this broader pattern. Importantly, accumulating evidence suggests that many of these MDR/XDR clones are likely introduced from external sources rather than arising locally. Accordingly, the key issue extends beyond the presence of resistance to the pathways by which these high-risk clones are introduced and disseminated. In particular, understanding how such clones enter Taiwan and subsequently move through the food chain is critical. Targeted investigation of transmission routes, especially those involving cross-border movement and food production systems, is therefore essential for effective control. Without such efforts, the continued introduction and spread of high-risk MDR/XDR *Salmonella* clones is likely to persist.

In conclusion, this study identifies a recent epidemiological shift of *S.* Kentucky in Taiwan driven by the emergence and expansion of AziR-XDR clone. Genomic analyses indicate that the predominant ST314 lineage likely originated from external sources, had undergone prior diversification, and subsequently expanded locally. The resistance phenotype is associated with a pre-assembled resistance architecture that is maintained through clonal spread rather than ongoing horizontal gene transfer. Together with previous findings on other serovars, these results highlight a recurring pattern in Taiwan in which high-risk antimicrobial-resistant *Salmonella* clones are repeatedly introduced and established. These findings emphasize the importance of strengthening surveillance and identifying the transmission routes by which such clones enter Taiwan and disseminate through the food chain. Addressing these pathways is essential to mitigate the continued emergence and spread of MDR/XDR *Salmonella*.

## Data Availability

The datasets presented in this study can be found in online repositories. The names of the repository/repositories and accession number(s) can be found in the article/Supplementary material.

## Footnotes

1
https://github.com/OpenGene/fastp

2
https://github.com/phac-nml/sistr_cmd

3
http://www.genomicepidemiology.org

4
https://github.com/tseemann/mlst

5
https://github.com/mikolmogorov/Flye

6
https://github.com/gbouras13/dnaapler

7
https://github.com/nanoporetech/medaka

8
https://www.ncbi.nlm.nih.gov/pathogens/

9
https://github.com/tseemann/snippy

10
https://github.com/nickjcroucher/gubbins

11
https://github.com/amkozlov/raxml-ng

12
https://itol.embl.de
