# Supplementary material for: Emergence of azithromycin-resistant extensively drug-resistant Salmonella Kentucky ST314 in Taiwan
Source: Front Microbiol. 2026 Jun 1;17:1832575. doi: 10.3389/fmicb.2026.1832575 (PMC13265453; doi:10.3389/fmicb.2026.1832575)
Supplement: Supplementary file 1 [file Data_Sheet_1.pdf]

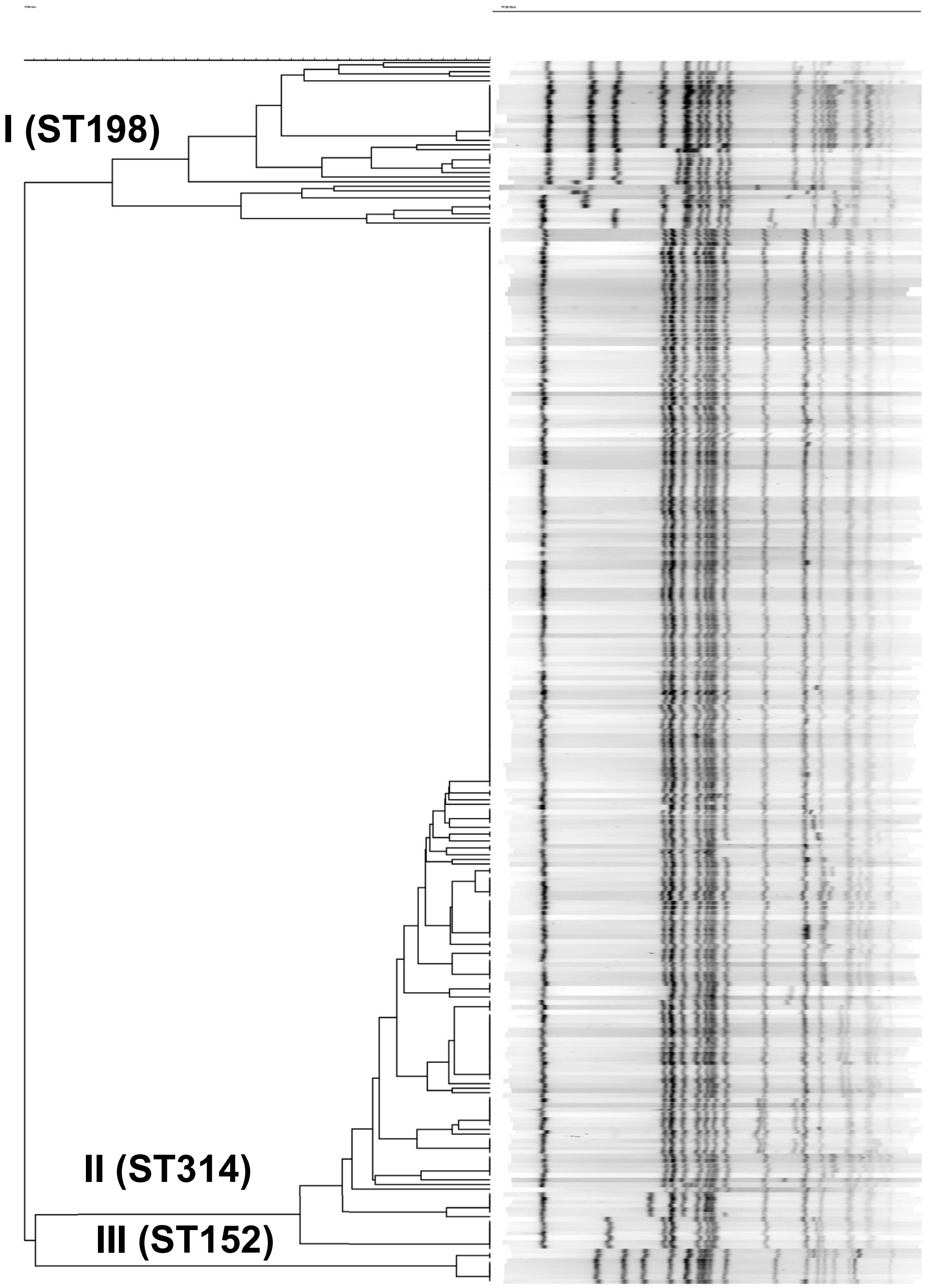

Figure S1. Dendrogram of 267 *S.* Kentucky isolates based on PFGE profiles. Analysis was performed using BioNumerics software (version 6.6) with the Dice similarity coefficient and clustering by the unweighted pair group methodwith arithmetic mean (UPGMA). Optimization was set at 1.5% and band position tolerance at 0.9%. Isolates were recovered from humans (n = 128), chickens (n = 32), chicken meat (n = 89), pigs (n = 15), clams (n = 2), and ducks (n = 1) between 2005 and 2024.

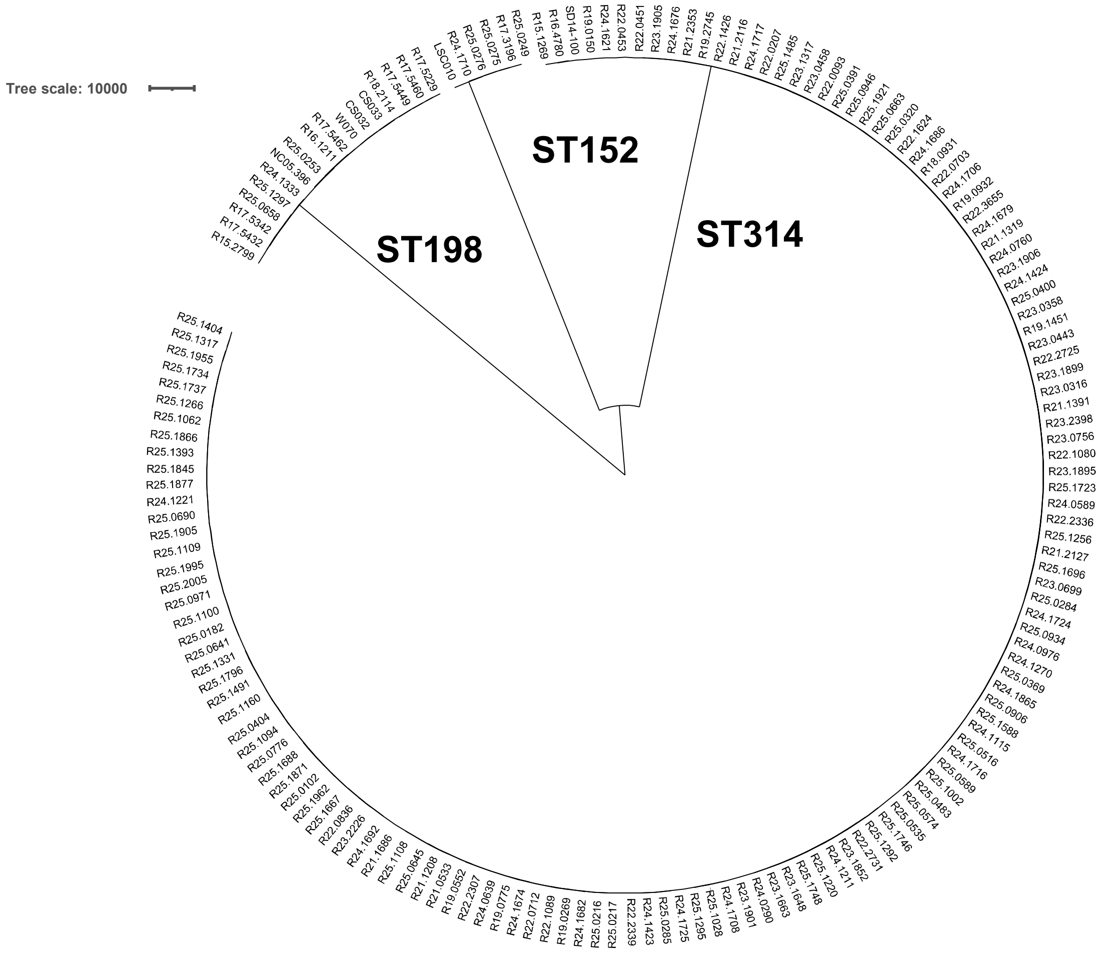

Figure S2. cgSNP-based phylogenetic tree of 165*Salmonella enterica* serovar Kentucky isolates. The maximum-likelihood tree was inferred from cgSNP profiles to assess genetic relatedness among isolates. Three major clades corresponding to sequence types (STs) 152, 198, and 314 were indicated. Scale bar indicates the number of SNP differences

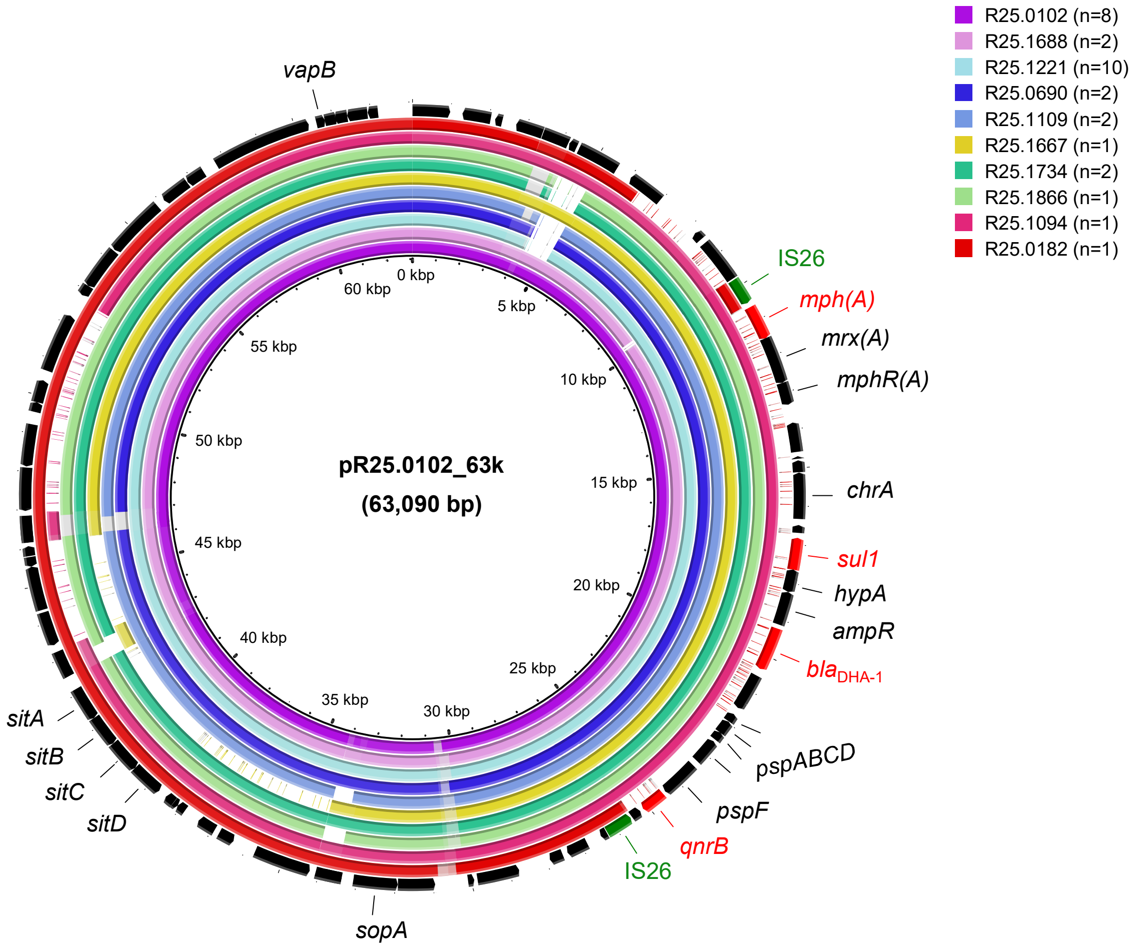

Figure S3. Comparative analysis of IncFIB(pB171) plasmids from 30 ST314 *Salmonella enterica* serovar Kentucky isolates using the BLAST Ring Image Generator (BRIG) with plasmid pR25.0102_63k as the reference sequence (1). The outermost ring represents the reference plasmid, and each concentric ring corresponds to an individual plasmid aligned against it. Colored regions indicate sequence similarity, whereas blank regions represent absent or highly divergent sequences. A total of 10 distinct plasmid structural patterns were identified among the 30 plasmids. Selected antimicrobial resistance genes, including *mph(A)*, *qnrB*, *sul1*, and *bla*$_{\text{DHA-1}}$, as well as the insertion sequence IS26, are indicated on the outer ring.

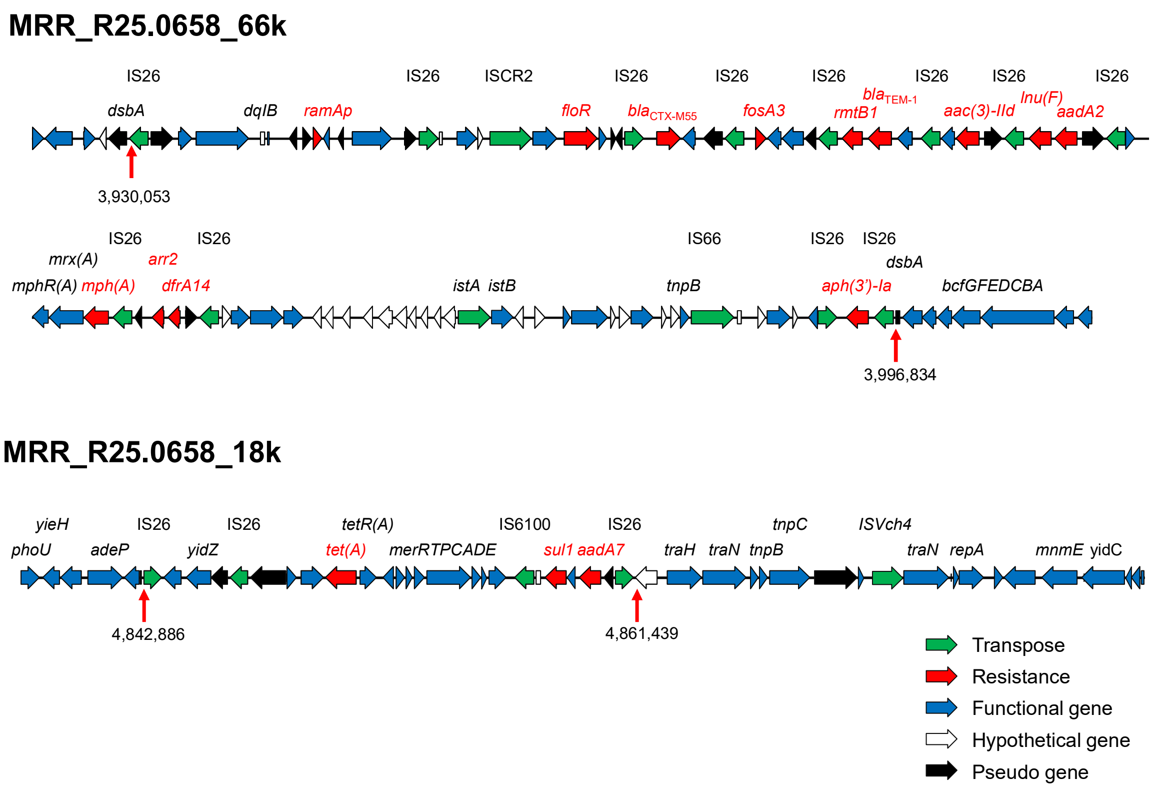

Figure S4. Genetic structure of the chromosomal multiple resistance regions (MRRs), MRR_R25.0658_66k and MRR_R25.0658_18k, in *Salmonella enterica* serovar Kentucky isolate R25.0658. Open arrows indicate predicted open reading frames and their transcriptional orientation. Antimicrobial resistance genes are highlighted in red and mobile genetic elements in green. Both MRRs are flanked by IS26 elements. MRR_R25.0658_66k is inserted within the *dsbA* gene, generating an 8-bp target site duplication. MRR_R25.0658_18k is also flanked by IS26 but lacks an identifiable target site duplication.

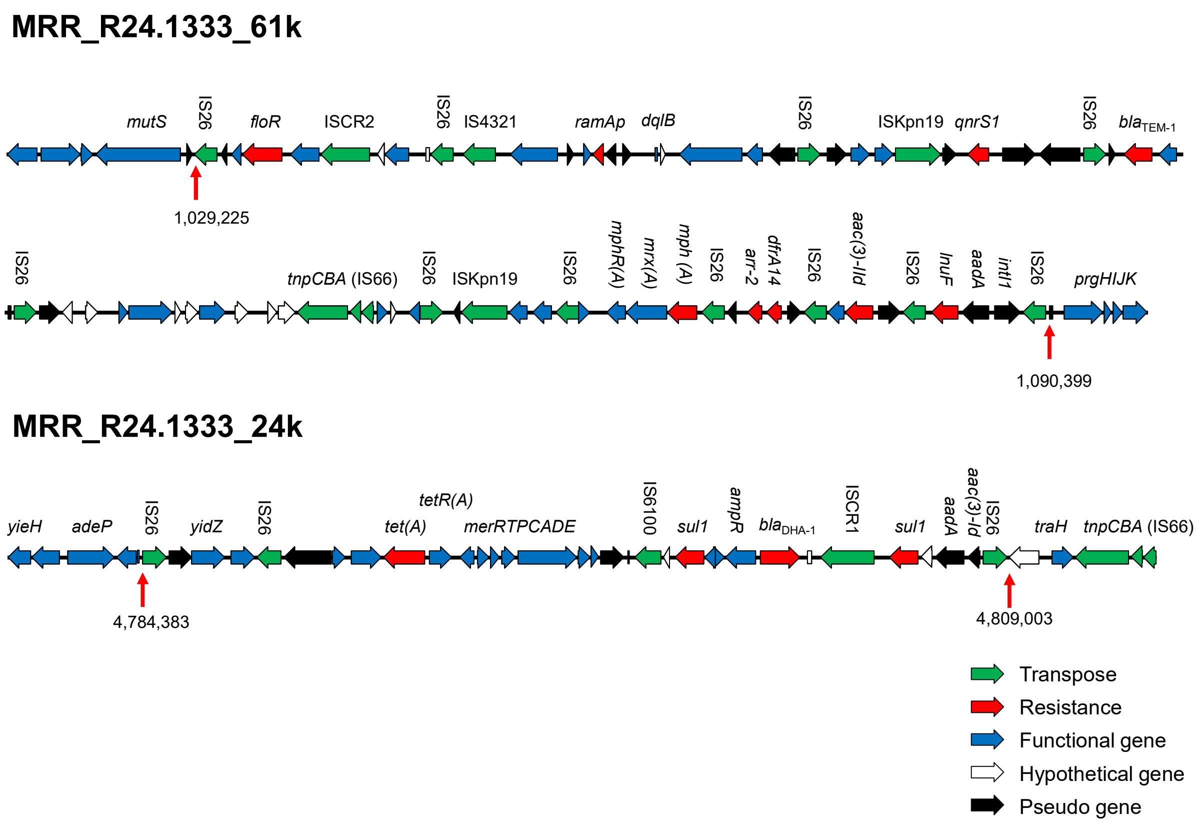

Figure S5. Genetic structure of the chromosomal multiple resistance regions (MRRs) in *Salmonella enterica* serovar Kentucky isolate R24.1333. Two chromosomal MRRs, designated MRR_R24.1333_61k and MRR_R24.1333_24k, are shown. Open arrows indicate predicted open reading frames and their transcriptional orientation. Antimicrobial resistance genes are highlighted in red and mobile genetic elements in green. Both MRRs are flanked by IS26 elements and lack an identifiable 8-bp target site duplication.

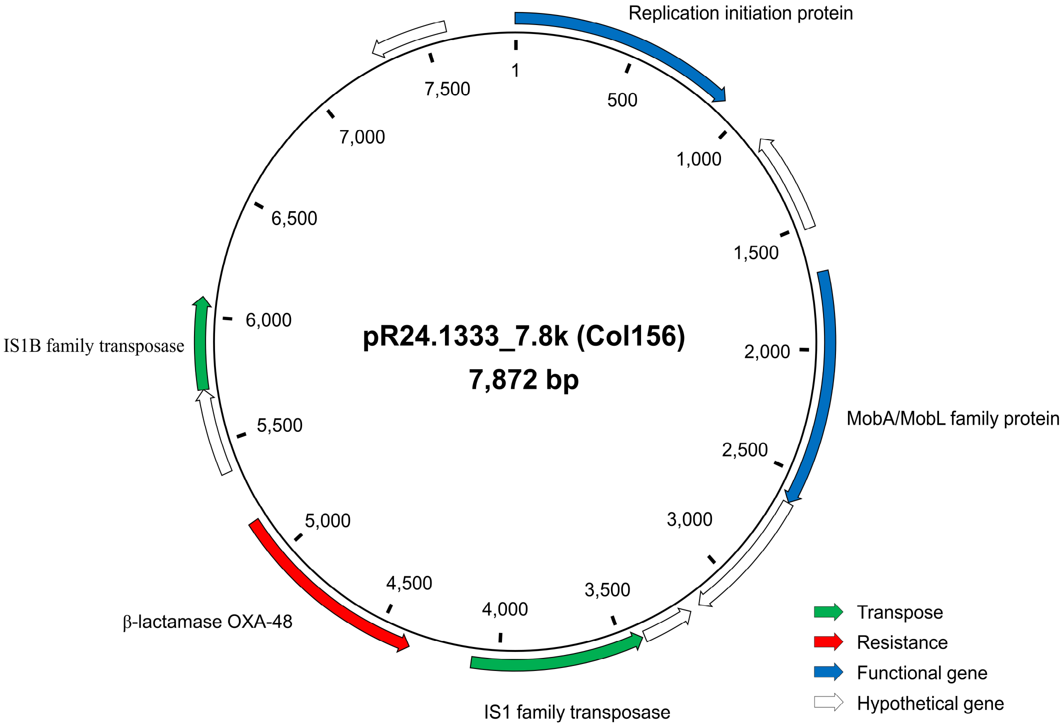

Figure S6. Genetic organization of the Col156 plasmid pR24.1333_7.8k carrying *bla*$_{\mathrm{OXA\text{-}48}}$. The circular map represents the complete sequence of the 7,872-bp plasmid pR24.1333_7.8k identified in *Salmonella enterica* serovar Kentucky isolate R24.1333. Arrows indicate predicted open reading frames and their transcriptional orientation. Antimicrobial resistance genes are shown in red and mobile genetic elements in green.

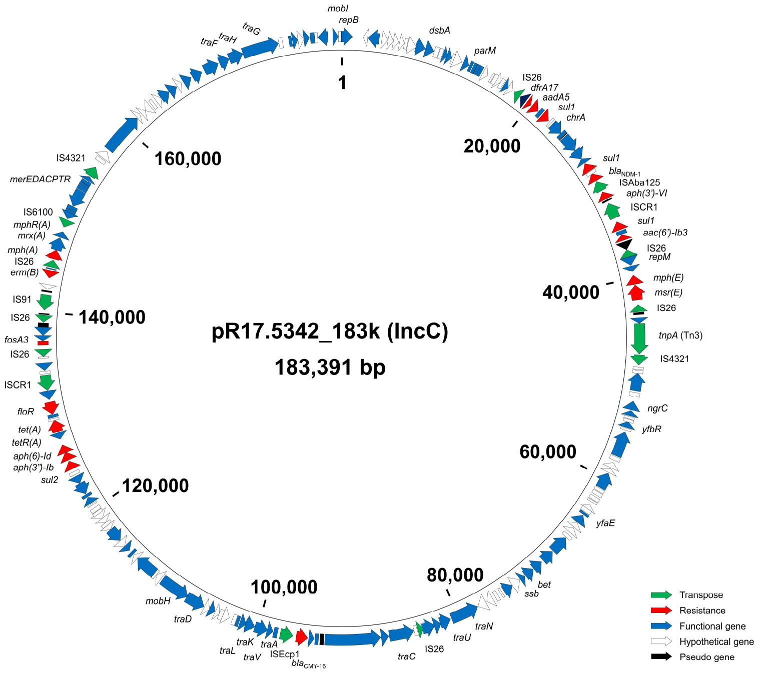

Figure S7. Genetic organization of the IncC plasmid pR17.5342_183k (183,391 bp) from *Salmonella enterica* serovar Kentucky isolate R17.5342. Arrows indicate predicted open reading frames and their transcriptional orientation. Antimicrobial resistance genes are shown in red and mobile genetic elements in green.

References

1. Alikhan NF, Petty NK, Ben Zakour NL, Beatson SA. 2011. BLAST Ring Image Generator (BRIG): simple prokaryote genome comparisons. BMC Genomics 12:402. http://dx.doi.org/10.1186/1471-2164-12-402.
